# SARS Coronavirus 3b Accessory Protein Modulates Transcriptional Activity of RUNX1b

**DOI:** 10.1371/journal.pone.0029542

**Published:** 2012-01-12

**Authors:** Bhavna Varshney, Sudhakar Agnihotram, Yee-Joo Tan, Ralph Baric, Sunil K. Lal

**Affiliations:** 1 Virology Group, International Centre for Genetic Engineering and Biotechnology, New Delhi, India; 2 Department of Microbiology and Immunology, University of North Carolina, Chapel Hill, North Carolina, United States of America; 3 Department of Microbiology, Yong Loo Lin School of Medicine, National University of Singapore, Singapore, Singapore; Centers for Disease Control and Prevention, United States of America

## Abstract

**Background:**

The causative agent of severe acute respiratory syndrome, SARS coronavirus (SARS-CoV) genome encodes several unique group specific accessory proteins with unknown functions. Among them, accessory protein 3b (also known as ORF4) was lately identified as one of the viral interferon antagonist. Recently our lab uncovered a new role for 3b in upregulation of AP-1 transcriptional activity and its downstream genes. Thus, we believe that 3b might play an important role in SARS-CoV pathogenesis and therefore is of considerable interest. The current study aims at identifying novel host cellular interactors of the 3b protein.

**Methodology/Principal Findings:**

In this study, using yeast two-hybrid and co-immunoprecipitation techniques, we have identified a host transcription factor RUNX1b (Runt related transcription factor, isoform b) as a novel interacting partner for SARS-CoV 3b protein. Chromatin immunoprecipitaion (ChIP) and reporter gene assays in 3b expressing jurkat cells showed recruitment of 3b on the RUNX1 binding element that led to an increase in RUNX1b transactivation potential on the IL2 promoter. Kinase assay and pharmacological inhibitor treatment implied that 3b also affect RUNX1b transcriptional activity by regulating its ERK dependent phosphorylation levels. Additionally, mRNA levels of MIP-1α, a RUNX1b target gene upregulated in SARS-CoV infected monocyte-derived dendritic cells, were found to be elevated in 3b expressing U937 monocyte cells.

**Conclusions/Significance:**

These results unveil a novel interaction of SARS-CoV 3b with the host factor, RUNX1b, and speculate its physiological relevance in upregulating cytokines and chemokine levels in state of SARS virus infection.

## Introduction

Severe acute respiratory syndrome (SARS) emerged in the Guangdong province of China in November 2002 and swept through more than 29 countries. Its spread infected more than 8000 people with a high mortality rate of 10%. It was found to be associated with a novel coronavirus named SARS-CoV [1], [2]. SARS-CoV, like other coronaviruses, is a positive sense, single-stranded enveloped RNA virus with a huge 29.7 Kb genome [3]. Its genome comprises of 14 ORFs which encode non-structural genes, structural genes and several unique group specific accessory proteins namely 3a, 3b, 6, 7a, 7b, 8a, 8b and 9b. [4]. Recognition of peptides derived from accessory proteins by convalescent sera of SARS-CoV infected patients [5] as well as their immuno-histochemical detection in infected VeroE6 cells and in clinical specimens [6] corroborates their expression during viral infection. However, these accessory proteins have been found dispensable for viral replication [7].

SARS-CoV accessory protein 3b is a 154 amino acid (aa) protein and has been characterized as one of the interferon antagonist encoded by SARS-CoV genome [8]. GFP tagged 3b has been reported to localize in the nucleus, nucleolus and mitochondria in cells [9], [10], [11]. A recent report delineated a unique nucleo-mitochondrial shuttling behaviour of 3b-GFP wherein 3b was found to inhibit RIG-I and MAVS induced type I interferon induction in the mitochondria [9]. Recently, we published a role of 3b in induction of AP-1 transcriptional activity that was mediated by the activation of ERK and JNK pathways [12]. Being an interferon antagonist that is dispensable for viral replication and observing its effect on the activity of crucial host transcription factors, 3b probably plays a role in disease progression by mediating viral-host interactions, which are poorly understood.

To uncover host interacting partners, of SARS-CoV 3b, we conducted a yeast two-hybrid screen of human lung cDNA library using 3b as bait. The screen identified RUNX1b (Runt related transcription factor 1, isoform b) as one of the host interacting partners of 3b. RUNX1 belongs to the RUNX family of genes which includes RUNX2 and RUNX3 additionally [13]. RUNX genes encode the α subunit, which heterodimerizes with the β subunit, CBFβ to form transcription factor CBF (Core Binding Factor) [14]. RUNX1 has a 128 aa runt domain through which it binds CBFβ as well as the consensus DNA element, TGT/cGGT [14], [15]. RUNX1 has three isoforms: RUNX1a, RUNX1b and RUNX1c. RUNX1a is a 250 aa protein with a runt domain. RUNX1b is a 453 aa protein and possess additional PST (proline, serine and threonine rich) region downstream to runt domain. RUNX1c differs from RUNX1b by 32 aa at N-terminus and is presumed to have similar functions in cells as RUNX1b [16]. RUNX1 is crucially required for definitive hematopoiesis and T-lymphocyte differentiation [17], [18]. At the molecular level, RUNX1 isoforms have been shown to regulate transcription of a number of genes including cytokines (IL2, IL3, GM-CSF etc.) and chemokines (MIP-1α, CSFR etc.). Based on the yeast two-hybrid screening results, we conducted our current study with the RUNX1b isoform. In this study, we confirmed the putative interaction of 3b and RUNX1b and observed *in vivo* recruitment of 3b on the RUNX1 binding element on the IL2 promoter in transiently transfected human T, jurkat cells. Further, 3b was found to increase the transactivation potential of RUNX1b on the IL2 promoter, which may partly be attributed to the enhanced ERK dependent phosphorylation of RUNX1b in 3b expressing cells. We next determined the positive effect of 3b-RUNX1b interaction on the expression of RUNX1b regulated chemokine MIP-1α, reported to be upregulated in SARS-CoV infected monocyte derived dendritic cells. Thus, we report a novel interaction of SARS-CoV 3b with RUNX1b and discussed its plausible significance in SARS virus pathogenesis.

## Results

### 3b interacts with RUNX1b

**Figure 1:**
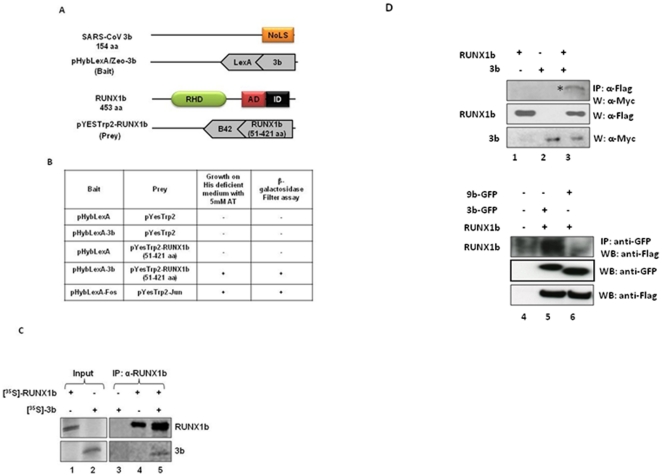
SARS-CoV 3b interacts with RUNX1b. A. A schematic representation of full-length 3b, pHybLexA/Zeo-3b bait plasmid, full-length RUNX1b and pYesTrp2-RUNX1b (51–421 aa) prey plasmid. The 3b protein has a nucleolar localization signal (NoLS) at the C-terminus. The RUNX1b protein has a runt homology domain (RHD, 50–177 aa), an activation domain (AD, 291–171 aa), and an inhibitory domain (ID, 346–411 aa). B. The 3b-RUNX1b interaction was assessed on a selective growth media (supplemented with 3-AT) and by filter lift β-galactosidase activity assay in a yeast two-hybrid experiment. pHybLexA/Zeo, pYesTrp2, pHybLexA/Zeo-3b, pYesTrp2-RUNX1b, pHybLexA/Zeo-Fos and pYesTrp2-Jun constructs were co-transformed in L40 in combinations tabulated above. pHybLexA/Zeo-Fos and pYesTrp2-Jun were used as positive control. C, D. *In vitro* analysis of 3b and RUNX1b interaction. C. *In vitro* translated S^35-^labelled 3b and RUNX1b lysates (input) were subjected to co-immunoprecipitation alone or together, using α–RUNX1 antibody. D. Total cell lysates of Huh7 cells expressing indicated proteins were immunoprecipitated with α-Flag antibody and western blotted with α-myc antibody to probe 3b protein (panel 1). Lysates were probed for the expression of RUNX1b and 3b with α-Flag and α-myc antibodies, respectively.

To identify cellular interacting partners of SARS-CoV accessory protein 3b, yeast two-hybrid screening of human lung cDNA library with full-length 3b as bait was conducted. The bait plasmid was constructed by cloning 3b coding sequence in-frame with the lex A DNA binding domain (Fig. 1A). The human lung cDNA library, cloned in-frame with the B42 activation domain vector was purchased commercially. From the screening, a clone encoding 51–421 aa of Runt related transcription factor 1, isoform b (RUNX1b, accession no. NP_001001890) (Fig. 1A) was obtained. Yeast co-transformants containing pYesTrp2-RUNX1b (51–421 aa) and pHybLexA/Zeo-3b withstood stringent growth conditions of media (supplemented with 5 mM 3-Aminotrizol) and scored positive for β–galactosidase filter lift assay (Fig. 1B) whereas co-transformants of pHybLexA/Zeo and pYesTrp2 (empty bait and prey plasmids); pHybLexA/Zeo-3b and pYesTrp2 (bait plasmid with empty prey plasmid); and pYesTrp2-RUNX1b and pHybLexA/Zeo (prey plasmid with empty bait plasmid) did not. pYesTrp2-Fos and pHybLexA/Zeo-Jun co-transformed yeast colonies were used as positive control.

RUNX1b and 3b physical interaction was confirmed *in vitro* by co-immunoprecipitation in two separate experiments. Firstly, co-immunoprecipitation using *in vitro* transcribed and translated full-length [^35^S]-3b and [^35^S]-RUNX1b showed pull-down of 3b by anti-RUNX1 from 3b-RUNX1b lysate mixture whereas no corresponding band for 3b was visible from the 3b and RUNX1b lysates alone (Fig. 1C). The interaction was further confirmed in Huh7 cells. Cells expressing flag tagged RUNX1b (pCMV-Tag2B Flag RUNX1b or Flag-RUNX1b) and myc tagged 3b (pCDNA 3.1 Myc/His-3b or Myc-3b), alone or together, were subjected to co-immunoprecipitation assay. Immunoprecipitation by anti-Flag detected 3b from cells co-expressing RUNX1b and 3b (Lane 3, Fig. 1D) whereas no corresponding band for 3b was seen from cells expressing RUNX1b or 3b alone (Lane 1 and 2, Fig. 1D). Reciprocal co-immunoprecipitation using anti-GFP antibody in cells expressing EGFP, 3b-EGFP, 9b-EGFP (negative control) and RUNX1b expression constructs showed pull-down of RUNX1b with 3b specifically, confirmed physical interaction of 3b with RUNX1b.

### 3b partially co-localizes with RUNX1b in the nucleus

**Figure 2:**
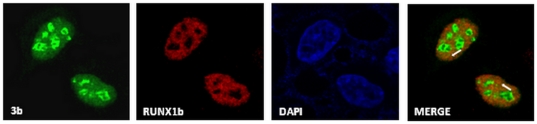
3b partially co-localizes with RUNX1b in the nucleus. Cellular distribution of 3b and RUNX1b proteins were visualized by subjecting Flag-RUNX1b and HA-3b transfected HEK293 cells to immunofluorescence assay. 3b was visualized using primary α–HA and alexa-488 conjugated secondary antibody. RUNX1b was visualized using primary α–RUNX1 and alexa-594 conjugated secondary antibody. Nuclei were visualized by DAPI (4′6-diamidino-2-phenylindole) staining. Arrows indicate extra nucleolar nucleus area of partial co-localization.

Cellular distribution of full-length 3b and RUNX1b was visualized in HEK293 cells using immunofluorescence assay. HEK293 cells transfected with Flag-RUNX1b and HA-3b (pXJ40-HA-3b) expression plasmids were subjected to immunofluorescence assay using anti-HA and anti-RUNX1 antibodies. Confocal microscopy revealed localization of RUNX1b in the nucleus, as reported earlier [19] and 3b in the nucleus with majority inside the nucleolus, as seen by Yuan et al [11]. Co-transfected cells expressing 3b and RUNX1b showed significant partial co-localization of the two proteins in the extra-nucleolar nucleus area with Pearson's correlation coefficient = 0.633 and Mander's coefficient = 0.9 (Fig. 2). Similar levels of partial co-localization were also seen in Cos7 cells (data not shown).

### 3b gets recruited on the RUNX1 binding element on the IL2 promoter

**Figure 3:**
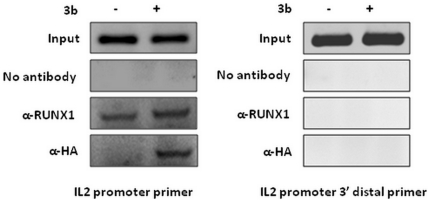
3b recruitment on RUNX1 binding elements on the IL2 promoter. Chromatin immunoprecipitation assays were performed with jurkat cells transfected with vector alone or HA-3b using α-RUNX1 and α-HA antibodies. PCR amplifications were performed using IL2 promoter primers and 3′ distal IL2 promoter primers. Results are representative of three independent experiments.

RUNX1b together with CBFβ forms CBF, which further interacts with other transcription factors, co-activators or co-repressors on RUNX1 binding elements and regulates transcription of target genes. The IL2 gene promoter harbours three RUNX1 binding sites and is reported to be regulated by RUNX1b in T cells [19]. To investigate whether 3b-RUNX1b interaction leads to the recruitment of 3b on RUNX1 binding elements on the endogenous IL2 promoter, ChIP assays were performed in RUNX1b/CBFβ endogenously expressing jurkat cells that are abortively infected by SARS-CoV. Jurkat cells transfected with empty vector or HA-3b were subjected to the assay after 48 h of transfection. ChIP results from 3b transfected cells using anti-HA depicted co-immunoprecipitation of the IL2 promoter region containing RUNX1 binding site but not from mock transfected cells (Fig. 3). However, the 3′-distal IL2 promoter region, which does not contain RUNX1 binding site, was not immunoprecipitated by anti-HA in either of the samples, suggesting that 3b recruitment on the IL2 promoter region is specific to the RUNX1 binding. Control ChIP assays using anti-RUNX1 (positive control) and no antibody (negative control) were conducted simultaneously. These results clearly demonstrate an *in vivo* recruitment of 3b on the RUNX1 binding element on the IL2 promoter.

### 3b increases RUNX1b transcriptional activity

**Figure 4:**
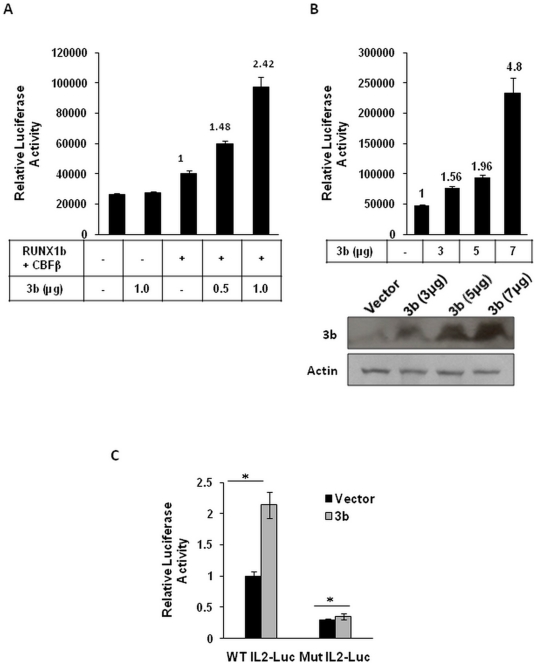
3b increases RUNX1b transactivation potential on the mouse IL2 promoter. A. HEK293 cells were transfected with WT IL2-Luc plasmid alone or with Flag-RUNX1b, Flag-CBFβ and indicated amounts of Myc-3b. Relative luciferase activities were calculated 48 h post transfection. B. Jurkat cells were transfected with WT IL2-Luc and indicated amounts of Myc-3b. Relative luciferase activities were calculated 48 h post-transfection. 3b expression was probed using α-myc antibody C. Jurkat cells were transfected with WT IL2-Luc or mutant IL2-Luc plasmid in the presence or absence of Myc-3b. Results in each panel are represented as mean±S.D. of triplicate cultures. Bar values represent fold increase in luciferase activity. *, *p*<0.005.

Viruses employ various strategies to manipulate activities of host transcription factors in their favour. To investigate the effect of SARS-CoV 3b protein on the RUNX1b transcriptional activity, reporter gene assays were performed using the mouse IL2 promoter. HEK293 cells were co-transfected with wild-type (WT) IL2 promoter luciferase plasmid, Flag-RUNX1b, Flag-CBFβ and Myc-3b in combinations mentioned. Renilla luciferase plasmid (pRL-TK) was co-transfected as an internal control. 3b alone showed no significant increase in the luciferase activity whereas 3b along with RUNX1b and CBFβ showed increase in the luciferase activity in a dose dependent manner (Fig. 4A). Transfection in jurkat cells also showed 1.5–5.0 fold increase in the luciferase activity with increasing doses of Myc-3b (Fig. 4B), suggesting that the increase in the luciferase activity in HEK293 and jurkat cells was specific to the 3b expression. To confirm whether 3b induced increase in the IL2 promoter activity was due to RUNX1b binding on the promoter, the IL2 promoter with all three RUNX1 binding sites mutated (mutant IL2-Luc) was used. Myc-3b transfected jurkat cells showed merely 0.3 fold increase in the luciferase activity with mutant IL2-Luc as against 2.0 fold with WT IL2-Luc (Fig. 4C); confirming that 3b dependent increase in the IL2 promoter activity relies on the RUNX1b transcription factor complex binding. Hence, we conclude that 3b potentiates RUNX1b transcriptional activity on the IL2 promoter.

### 3b stimulates RUNX1b activity by inducing its ERK dependent phosphorylation

**Figure 5:**
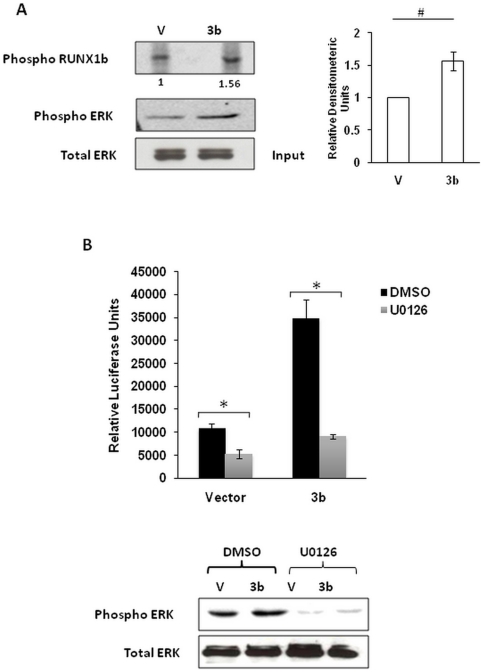
3b expression increases phosphorylated RUNX1b levels through ERK activation. A. HEK293 cells were transfected with vector, 3b and RUNX1b expression plasmids. ERK immunoprecipitated from vector and 3b lysates were subjected to kinase assay with RUNX1b beads. Phosphorylated RUNX1b was visualized by autoradiography. Input levels of immunoprecipitated ERK and phospho ERK levels in lysates were probed by western blotting. Graph depicts fold increase in the levels of phosphorylated RUNX1b procured after three independent experiments. Bar represents mean±SD of values obtained by densitometry. #, *p*<0.05. B. Jurkat cells were transfected with WT IL2-Luc in the presence or absence of Myc-3b and treated with DMSO or U0126. Relative luciferase activity was measured and is shown as the mean±SD of three independent experiments performed in triplicates. *, *p*<0.005. Phospho ERK levels in lysates were probed by western blotting.

RUNX1b transcriptional activity is regulated by various post-translational modifications like phosphorylation, acetylation, ubiquitination etc [20]. Phosphorylation of RUNX1b by Ser-Thr kinases/ERK on serine 249/266 has been reported to potentiate its transactivation potential [21]. SARS-CoV 3b has been reported to activate ERK pathway [12]. Therefore, determination of ERK dependent phosphorylation levels of RUNX1b in 3b expressing cells was of our interest. To investigate this, an *in vitro* kinase assay with 3b and vector transfected HEK293 cells was performed. RUNX1b immunocomplex from transfected cells served as a substrate for the kinase assay. Results depicted a 1.5 fold increase in phosphorylated RUNX1b levels in 3b transfected cells compared to control transfected cells (Fig. 5A). This suggests that 3b activated ERK may partly be responsible for the stimulated RUNX1b transcriptional activity in 3b transfected jurkat cells. To test this hypothesis, the reporter gene assay was performed in the presence of ERK inhibitor, U0126. 3b transfected jurkat cells were treated with DMSO or U0126 for 24 hrs prior to measurement of luciferase activity. Treatment of cells with U0126 led to the inhibition of luciferase activity which was otherwise observed in 3b expressing DMSO treated cells (Fig. 5B). This experiment points out that 3b mediated increase in RUNX1b transactivation potential may also be contributed by stimulated ERK activity.

### 3b and RUNX1b cooperatively enhance MIP-1α mRNA levels

**Figure 6:**
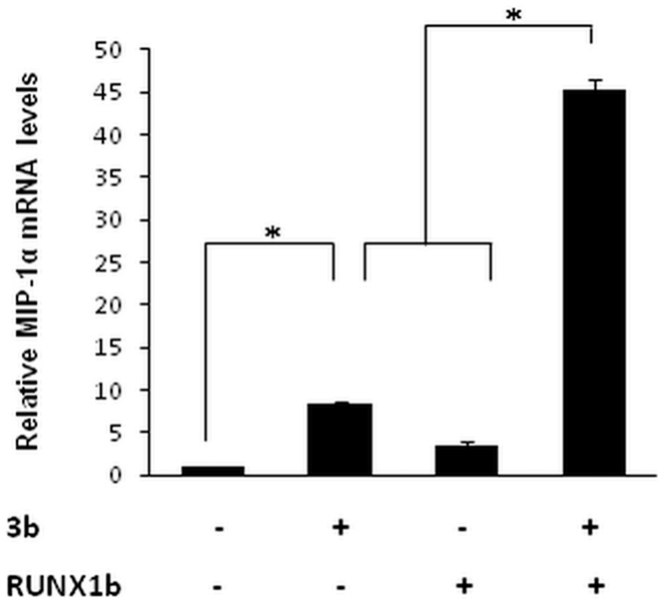
3b and RUNX1b cooperatively increase MIP-1α mRNA levels. Relative mRNA levels of MIP-1α to actin were estimated in U937 cells expressing indicated proteins, using quantitative RT-PCR. Histogram is the result of three independent experiments. Bar values represent fold increase in the mRNA levels. Asterisk *, *p*<0.005.

Promoter of macrophage inflammatory protein (MIP-1α), a well characterised pro-inflammatory cytokine [22] have been documented to be regulated by RUNX1b through its proximal and distal RUNX1 binding elements [23]. SARS-CoV infection has been found to stimulate monocyte derived-dendritic cells to express MIP-1α [24]. In order to study the effect of 3b expression on endogenous MIP-1α mRNA levels, real-time PCR was performed in human monocytic U937 cells. Cells overexpressing RUNX1b showed 3.0 fold increase in MIP-1α mRNA levels; whereas those expressing 3b showed 5.0 fold increase as compared to vector. Interestingly, cells overexpressing 3b along with RUNX1b showed 13 fold increase in MIP-1α mRNA levels (Fig. 6), suggesting that 3b significantly enhances RUNX1b transactivation potential on the endogenous MIP-1α promoter.

## Discussion

SARS pathogenesis, caused by evasion of the host innate immunity, is characterized by a remarkable cytokine storm and lymphopenia. SARS coronavirus antagonizes host interferon action by encoding a few interferon antagonists like 3b, SARS 6, nucleocapsid, nsp1, nsp3 and recently reported, M protein [8], [25], [26]. Among them, 3b, like SARS6, belongs to a group of accessory proteins that are unique to SARS coronavirus. A recent study brought out plausible contribution of 3b in SARS pathogenesis, by affecting levels of chemokines that are found upregulated in SARS. We believed that the accessory protein 3b has more roles to play in virus pathogenesis which are still unknown. To unveil new cellular interactors of 3b, we employed a yeast two-hybrid screen of human lung cDNA library and identified transcription factor RUNX1b as a putative interactor of 3b. The interaction was validated *in vitro* by co-immunoprecipitation.

RUNX1b plays a crucial role in development of myeloid and lymphoid lineage cells. It was originally identified at a break point on human chromosome 21 in the t(8;21) translocation. It is a most common target of chromosomal translocation in human leukaemias [16], [27] including acute myeloid leukaemia, B-cell acute lymphoblastic leukaemia and T-cell lymphoblastic leukaemia [28], [29]. Importantly, RUNX1b is involved in transcriptional regulation of several genes including cytokines and chemokines like IL2, IL3, GM-CSF, MIP-1α, CSFR etc. [19], [23], [30], [31], [32], [33], [34], [35]. It synergises with other transcription factors to activate transcription [36], [37], [38] and acts as a transcriptional activator or repressor depending upon its association with co-activators such as CBP, p300, MOZ [39], [40] and co-repressors, mSin3A [41], TLE1 [42], [43] and NCoR [28].

Several reports have described the involvement of RUNX1 isoforms in transcription and replication of viruses like murine leukaemia virus [14], [44], [45], maedi visna virus [46], polyomavirus [47] and human papilloma virus [48], [49]. B-cell proliferation and immortalization by Epstein-Barr virus is mediated by downregulation of RUNX1, a consequence of the binding of RUNX3 on the RUNX1 promoter [50]. RUNX1 has been found to be upregulated in latently infected GM-Ps by human cytomegalovirus (CMV), a species specific herpesvirus [51]. In adenovirus infected cells, RUNX1 leads to significant mislocalization of E4Orf6, thus interfering with viral replication [52]. A comparative study by microarray analysis of host gene transcription in the Huh7 cell line infected with SARS-CoV and human coronavirus 229E found significantly increased RUNX1 expression with SARS-CoV infection as compared to human CoV22E [53]. This observation prompted us to study this novel interaction of SARS-CoV accessory protein 3b with RUNX1.

We observed significant partial co-localization of 3b and RUNX1b in the extra-nucleolar nucleus region. Next, 3b was found to get recruited on the RUNX1 binding elements which led to increased transactivation of RUNX1b on the IL2 promoter, as depicted by reporter gene assays. Additionally, 3b also affected its transactivation on the IL2 promoter by modulating phosphorylation levels of RUNX1b through ERK. A similar mode of regulation of immediate early gene X-1 through ERK-induced RUNX1 phosphorylation in response to thrombopoetin was reported by Hamelin and colleagues [54]. In order to explore the effect of 3b on RUNX1b target gene other than IL2, we measured mRNA levels of a MIP-1α, the promoter of which is reported to be regulated by RUNX1b [23]. MIP-1α has been observed to get upregulated in monocyte derived dendritic cells infected with SARS-CoV [24]. In our study, overexpression of 3b in monocytic cells U937 resulted in 5 fold increase in MIP-1 mRNA levels which rose to 13 folds, when overexpressed with RUNX1b. Interestingly, a recent report by Chen et al. characterizing cellular immune response to SARS-CoV infection in senescent mouse models showed increase in MIP-1α and IL2 levels at early and late stages of infection [55], a result that supports our observations.

To the best of our knowledge, this is the first report unveiling the effect of SARS-CoV protein 3b on the transcriptional activity of a host transcription factor, RUNX1b, and its downstream target genes IL2 and MIP-1α. Jurkat and monocyte cells are abortively infected by SARS-CoV. Upon entry in these cells, RNA genome of the SARS-CoV will get transcribed and translated and expectedly, would lead to the synthesis of 3b along with other proteins. However, until now there is no direct report on the expression levels of 3b in these virus infected cells. Therefore, based on our study we have speculated the role of 3b in virus infected monocytic and T cells and put forth a plausible role of 3b in SARS-CoV pathogenesis which gives new directions to the understanding of SARS.

## Materials and Methods

### Reagents and plasmids

3-Aminotrizole was purchased from Sigma. Luciferase assay kit (E1500) and *in vitro* transcription and translation kit (L4610.) were purchased from Promega. TRIzol was purchased from Invitrogen. Antibodies against RUNX1, HA and c-Myc were purchased from Santa Cruz. Anti-Flag was purchased from Roche. Alexa 488 conjugated anti-mouse and alexa 594 conjugated anti-rabbit were purchased from Molecular Probes. The SARS-CoV 3b gene (25689–26153 bp), was PCR amplified from the SARS-CoV genome (NC_004718) and cloned in pXJ40-HA vector as described earlier [56]. The 3b insert was further cloned into pHybLexA/Zeo and pCDNA3.1(-) myc/his vector. pCMVFlag Tag 2B-RUNX1b, mouse WT IL2-Luc and mutant IL2-Luc (all three RUNX1 binding sites mutated) constructs were generously provided by Dr. Shimon Sakaguchi (Institute for frontier Medical Sciences, Kyoto University, Japan), MigRI-CBFβ was gifted by Dr. Nancy A Speck (University of Pennsylvania School of medicine, Philadelphia).

### Yeast Two-hybrid screening

Lex A based screening system (Hybrid hunter, version F), comprised of *Saccharomyces cerevisiae* strain L40 [*MATa his3*Δ*200 trp1-901 leu2-3112 ade2 LYS2::(*4*lex*Aop-*HIS3)URA3*::(8*lex*Aop-*lac*Z) *GAL4]*, pHybLexA/Zeo and pYesTrp2 as binding domain and activation domain vectors, respectively and human lung cDNA library cloned in pYesTrp2, was purchased from Invitrogen. Screening was performed as per manufacturer's protocol. The bait plasmid was constructed by cloning 3b coding sequence in-frame with the LexA DNA binding domain in pHybLexA/Zeo. pHybLexA/Zeo-3b was co-transformed with cDNA library in L40 and co-transformants were selected for the activation of two reporter genes, *HIS3* and *LacZ*. Strength of the interaction in selected co-transformants were assessed by their ability to grow on His^−^ Trp^−^ and Zeo^+^ YC media supplemented with 5 mM AT (3-amino-1,2,3-trizole, competitive inhibitor of HIS3) and for positivity of filter β–galctosidase activity assay. Filter-lift assay was performed as described before [57]. Plasmids were isolated from positive co-transformants and shuttled into *E.Coli* DH5α and sequenced. L40 co-transformed with pHybLexA/Zeo-Fos and pYesTrp2-Jun was used as the positive control and L40 co-transformed with pHybLexA/Zeo and pYesTrp2 was used as the negative control for the library screening.

### Cell culture and transfection

HEK293, human embryonic kidney cells and Huh7, human hepatoma cells were maintained in DMEM (Dulbecco's modified Eagle's medium). Human leukemic T cells, jurkat and human monocytic cells, U937, were maintained in RPMI1640. All cell lines were maintained in media supplemented with 10% FCS (fetal calf serum, v/v) and antibiotics (penicillin and streptomycin). Transient transfections in Huh7 and HEK293 cells were carried out using fugene 6 (Roche) and lipofectamine 2000 (invitrogen) as per manufacturer's protocol and in jurkat and U937 cells were carried out by electroporation at 260 V and 975 µF in 4 mm cuvettes, using Biorad Gene Pulser.

### Immunofluorescence assay

For immunofluorescence assay, HEK 293 cells were seeded at 50% confluency on coverslips in a 12 well plate. Cells were singly or co-transfected with RUNX1b (pCMV-Tag2B Flag RUNX1b) and 3b (pXJ40 HA-3b) expression vectors and the assay was performed 24 h post-transfection as described by Korkaya et al. [58]. 3b and RUNX1b was stained using mouse anti-HA and rabbit anti-RUNX1 antibodies, respectively. Alexa 594 conjugated goat anti-rabbit and alexa 488 conjugated goat anti-mouse were used as secondary antibodies. Coverslips were mounted on to the glass slides in anti-fade (with DAPI) medium. Confocal images were collected using a 60× objective on a Nikon A-1Rconfocal microscope and analysed by imaging software, NIS-elements.

### Co-immunoprecipitaion and Western blotting

For *in vitro* co-immunoprecipitation, [^35^S] radiolabelled full length RUNX1b and 3b were expressed using a coupled *in vitro* transcriptional/translation system as per manufacturer's protocol. 3b lysate, RUNX1b lysate and 3b-RUNX1b lysate mix were incubated with anti-RUNX1 in 500 µl lysis buffer (20 mM Tris pH 7.4, 150 mM NaCl, 0.05% NP40) at 4°C for 4–6 h. Immunocomplexes were precipitated by adding 50 µl sepharose-A (50% v/v) beads at 4°C for 1 h. Beads were washed thrice with lysis buffer and resuspended in SDS-PAGE loading buffer. Samples were run on SDS-PAGE and bands were detected by autoradiography. For *in vivo* co-immunoprecipitaion, Huh7 cells were transiently transfected with expression plasmids of RUNX1b (pCMV-Tag2B Flag RUNX1b) and 3b (pCDNA3.1 Myc-3b) in combinations mentioned. Total DNA amount was normalized by adding pCDNA3.1. 48 h post transfection, cells were lysed and immunoprecipitation and western blotting was performed as described earlier [59].

### Chromatin immunoprecipitation (ChIP)

For ChIP, 10–15×10^6^ Jurkat cells were transfected with 10 µg vector or 3b (pXJ40 HA-3b) plasmid. 48 h post transfection, ChIP assay was performed as described earlier [60]. DNA fragments were analysed for the recruitment of RUNX1b and 3b on the RUNX1 binding site on the IL2 promoter as well as on 5′ distal region of the human IL2 gene (non-RUNX1 binding site). Sequences of the primers used for PCR: forward primer for human IL2 promoter: 5′-CTCTAGCTGACATGTAAGAAGC-3′; reverse primer for human IL2 promoter: 5′-CTACACTGAACATGTGAATAGC-3′; Forward primer for 3′ distal region of the human *IL-2* gene: 5′-AAATGTTGCAGGATCCTTGC-3′; reverse primer for 3′ distal region of the human *IL-2* gene: 5′-TGAGCTCTGACATGATGCTC-3′.

### Luciferase assay

Luciferase assay was performed as per manufacturer's protocol (Promega). For the assay, cells were transfected with reporter plasmid (WT IL2-Luc or mut IL2-Luc), pRL-TK and indicated expression plasmids. pEGFP-N1 plasmid was transfected as a negative control in cells not expressing 3b. Drug treatment (U0126, 10 µM) was performed for 24 h prior to harvesting. All transfections were normalized for the amount of total DNA by adding pCDNA3.1. Luciferase activity was measured 48 h post transfection and normalized against renilla luciferase activity. Relative luciferase activity was expressed as mean±standard deviation (SD) of three independent experiments. Statistical significance was calculated using student's t test. *p* values are given in the figure legends. Error bars represent the SD.

### *In vitro* kinase assay

ERK activity in 3b transfected cells was assayed using immunoprecipitated RUNX1b as the substrate. HEK293 cells were transfected with expression plasmids of vector (control), 3b, and RUNX1b. 48 h after transfection, cells were lysed in lysis buffer (20 mM Tris-HCl pH 7.5, 150 mM NaCl, 1 mM EDTA, 1 mM EGTA, 1% Triton, 2.5 mM sodium pyrophosphate, 1 mM β-glycerophosphate, 1 mM sodium orthovandate) and equal amounts of vector and 3b lysates were subjected to immunoprecipitaion using anti-ERK antibody and RUNX1b lysate, using anti-RUNX1 antibody. Sepharose A beads were washed thrice with the lysis buffer and twice with the kinase buffer (20 mM Tris–Cl pH 7.5, 10 mM MgCl_2_, 1 mM DTT, 5 mM Sodium orthovandate). For the assay, ERK beads were incubated with RUNX1b beads in 20 mM Tris–Cl (pH 7.5), 10 mM MgCl_2_, 1 mM DTT, 2 mM β-glycerophosphate and 50 mM ATP, 5 mCi [γ-^32^P]-ATP at 37°C for 30 min. Reaction mixtures were run on SDS–PAGE, and analyzed by autoradiography.

### RNA isolation and real time PCR

RNA was isolated from transfected U937 cells using TRIzol, as per manufacturer's protocol. 5 µg RNA was reverse transcribed using oligo-dT primer and MuLV reverse transcriptase as per manufacturer's protocol (Promega). Primers used for PCR were: MIP-1α forward: 5′ ACTTGCTGCTGACACGCCGA 3′ and reverse: 5′ CACAGACCTGCCGGCTT CGC 3′; Actin forward: 5′ TGACGGGGTCACCCACACTGTGCCCATCTA3′ and reverse: 5′ CTAGAAGCATTTGCGGTGGACGATGGAGGG3′. Data is represented as bar graph and is mean±SD of three independent observations.
