# Correction: SARS Coronavirus 3b Accessory Protein Modulates Transcriptional Activity of RUNX1b

**DOI:** 10.1371/annotation/64ae6047-0f9b-4d17-a065-e08c153aa435

**Published:** 2012-03-07

**Authors:** Bhavna Varshney, Sudhakar Agnihothram, Yee-Joo Tan, Ralph Baric, Sunil K. Lal

There was an error in the second author's name. The correct spelling is Sudhakar Agnihothram. The correct citation is: Varshney B, Agnihothram S, Tan Y-J, Baric R, Lal SK (2012) SARS Coronavirus 3b Accessory Protein Modulates Transcriptional Activity of RUNX1b. PLoS ONE 7(1): e29542. 

